# Extracellular matrix and synapse formation

**DOI:** 10.1042/BSR20212411

**Published:** 2023-01-06

**Authors:** Lei Yang, Mengping Wei, Biyu Xing, Chen Zhang

**Affiliations:** School of Basic Medical Sciences, Beijing Key Laboratory of Neural Regeneration and Repair, Advanced Innovation Center for Human Brain Protection, Capital Medical University, Beijing 100069, China

**Keywords:** extracellular matrix, neuroscience, Synapse formation, synaptic plasticity, synaptogenesis

## Abstract

The extracellular matrix (ECM) is a complex molecular network distributed throughout the extracellular space of different tissues as well as the neuronal system. Previous studies have identified various ECM components that play important roles in neuronal maturation and signal transduction. ECM components are reported to be involved in neurogenesis, neuronal migration, and axonal growth by interacting or binding to specific receptors. In addition, the ECM is found to regulate synapse formation, the stability of the synaptic structure, and synaptic plasticity. Here, we mainly reviewed the effects of various ECM components on synapse formation and briefly described the related diseases caused by the abnormality of several ECM components.

## Introduction

A *synapse* is a structure in which information is transmitted from one neuron to another neuron or target cell. A typical synapse comprises the presynaptic compartment, synaptic cleft, and postsynaptic compartment [1]. The presynaptic terminal mainly contains an active zone, which is the transmitter release site, and synaptic vesicles are clustered around the active zone [2,3]. In contrast, the postsynaptic compartment mainly contains neurotransmitter receptors and downstream signaling protein complexes [4–6]. The synaptic cleft is a 20-nm cleft formed by the pre- and post-synaptic membrane. Plenty membrane/secretory proteins are reported to be located in the synaptic cleft to maintain the synaptic structure and signaling transduction. Through the assembly and stabilization of the presynaptic, postsynaptic, and synaptic cleft proteins, the synaptic structures are formed and matured, and functional connections between neurons are established [7].

Synapse formation is a complicated and dynamic process, including the development of axons and dendrites, pre- and postsynaptic organization and connection, and synapse remodeling [8,9]. In all these processes, the pre- and postsynaptic component organization and connection are the core processes that determine functional synapse formation. Synapse formation occurs not only during organisms’ development but also throughout its lifespan [10,11]. Given the vast numbers and diverse types of synapses, the mechanisms of synapse formation remain unclear. In the past few years, plenty molecules have been discovered that regulate synapse organization. The most well-examined factors are cell adhesion molecules (CAMs) [12–14]. CAMs organize synaptic connections through trans-synaptic protein–protein interaction and stabilize/destabilize the synapse under different circumstances [15,16]. The class of secretory factors also organizes synapses [17,18]. Secretory factors are released by neurons or glia. They constitute the extracellular matrix (ECM) around synapses and participate in the development and formation of synapses [19–21].

**Figure 1:**
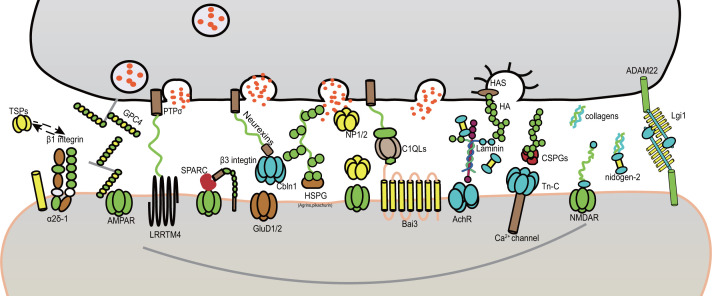
Components of the extracellular matrix are involved in regulating synapse formation

In the mammalian brain, the ECM, which comprises approximately one-fifth of the brain's volume, is required for maintaining the nervous system’s structure and signal transduction [22]. The components of the ECM are synthesized by glial cells and neurons in different proportions and are wrapped around cells [23]. The mechanical homeostasis of ECM is important for tissue-level structural integrity [24]. Furthermore, the signal transduction function of the ECM is mainly performed via interactions between various components of the ECM. The ECM can activate cell surface receptors through interactions with receptors [25]. In recent years, much research has reported that the ECM plays a critical role in the development and maturation of the nervous system via the regulation of nerve cell migration, neurite outgrowth, synaptogenesis, synaptic plasticity, and synaptic structure stabilization [26]. During synapse formation and maturation, ECM components and their receptors penetrate the synaptic cleft (Figure 1) and play critical roles as guide molecules, helping neurons send axonal and dendritic projections, establish connections, and organize the presynaptic and postsynaptic compartment [27]. After stable synapses are formed, the roles of several ECM receptors shift to control the maintenance of synaptic structures, stabilize connections, and regulate synaptic plasticity [28,29]. In this review, we introduce structural and non-structural ECM components, including thrombospondins (TSPs), glypicans, SPARC/hevin, neuronal pentraxins, C1q-like (C1QL) proteins, precerebellin, Lgi1, agrins, laminins, collagens, nidogens, pikachurin, tenascin, hyaluronan, reelin, chondroitin sulfate proteoglycans, and matrix metalloproteinases, which are reported to play a role in stabilizing the structures of synapses, inducing pre- and postsynaptic specialization and regulating synaptic transmission and synaptic plasticity.

## ECM components serving as synaptic organizers

### Thrombospondin

TSPs are defined as prototype matricellular proteins that are nonstructural ECM components and play a critical role in ECM assembly [30]. The functions of TSPs are based on interactions with ligands, such as the structural components of ECM, receptors, growth factors, cytokines, and proteases [31]. TSPs have been found to play a role in cell–cell and cell–matrix interactions during wound repair and tissue remodeling [32,33]. The ablation of TSPs causes abnormalities in connective tissue development (such as ligaments and tendons) [34] and abnormalities in angiogenesis during wound healing [33,35]. There are five types of TSPs (TSP1–5). All five members of the family exhibit synaptogenesis activity [36,37]. TSPs are expressed and released from astrocytes [38]. TSP1 and TSP2 are trimeric proteins, while TSP3, TSP4, and TSP5 are tetrameric proteins [39,40]. TSP1, TSP2, and TSP3 are mainly expressed in immature astrocytes; few are expressed in adult astrocytes. In contrast, TSP4 is only expressed in adult astrocytes and controls synapse formation and synaptic plasticity in the adult brain [36]. The best-examined TSPs in synapse formation are TSP1 and TSP2. Retinal ganglion cells (RGCs) cultured in TSP1- and TSP2-containing mediums exhibit more synaptic puncta compared with the control medium, indicating that TSP1 and TSP2 can promote the synapse formation of RGCs *in vitro* [39]. However, the number of synaptic proteins in RGCs cultured with TSP1-containing medium has not increased considerably, indicating that the effect of TSP1 on synapse formation does not depend on the synthesis of new synaptic proteins [39]. The ultrastructure of induced synapses is normal and displays presynaptic activity, whereas the loss of glutamate receptors in the postsynaptic membrane leads to postsynaptic silence [41]. In addition, in TSP1 and TSP2 double knockout mice, the number of excitatory synapses was significantly reduced. Studies have elucidated the mechanism by which TSPs promote synapse formation. People have identified that the non-pore-forming auxiliary subunit of calcium channels α2δ-1 is a binding partner for TSPs [40]. As the receptor of TSP, it can mediate the formation of synapses by interacting with the epidermal growth factor-like repeats of TSPs. After the nervous system is damaged, the expression of TSP and α2δ-1 increase. Thus, they play an important role in synaptic remodeling after injury. Previous research has demonstrated that GBP can block the interaction between TSP and α2δ-1. Hence, gabapentin (GBP) can be used to block the excessive formation of synapses and resist nerve pain [40]. In addition, as a potential ligand for β1 integrin, TSP1 can slow the movement of extrasynaptic glycine receptors in spinal cord inhibitory synapses and stabilize such receptors in synapses [42]. In summary, TSP plays a vital role in synapse formation, synaptic development, synaptic function, and synaptic remodeling after injury [43].

### Glypican (GPC) 4/6

GPCs are a member of the heparan sulfate proteoglycan (HSPG) family with a glycosyl phosphoinositide (GPI) anchor [44]. HSPGs are components of ECM [45–47] and play a role in cell–cell and cell–matrix interactions and cellular signaling transduction in the ECM [47]. GPCs are considered matricellular proteins, and they can shape the extracellular matrix and are involved in ECM remodeling [48,49]. GPCs are often highly glycosylated and tether to the plasma membrane with the GPI anchor [50]. The GPI anchor can be cleaved by phospholipases, and then GPCs can be released into the extracellular space [51]. GPCs in mammals have six members: GPC1–6. Among the six members, GPC4 and 6 are highly expressed in the central nervous system (CNS) [52]. GPC4 is expressed in the hippocampus, whereas GPC-6 is expressed in the cerebellum. GPCs in the CNS are expressed by the glia and released into the extracellular environment [53]. An astrocyte-conditioned medium with GPC4 and 6 can induce synapse formation in cultured retinal ganglion cells [53]. Unlike TSPs, synapses induced by GPCs are functional, exhibiting increased surface expression levels of α-amino-3-hydroxy-5-methyl-4-isoxazole propionic acid (AMPA) receptors [54]. The depletion of GPC4 in mice decreases the amplitude of AMPAR-mediated excitatory synaptic currents without changing the number of synapses [53]. GPCs can interact with presynaptic RPTPδ and induce the release of neuronal pentraxin from axons. Pentraxin, which will be discussed in Section 4, can recruit and maintain AMPA receptors in the neuronal surface [54]. GPCs can bind to several synaptic organizing CAMs. Two research groups have reported that leucine-rich repeat transmembrane neuronal protein 4 (LRRTM4), a transmembrane protein with synaptogenesis activity, interacts with GPC4 [55,56]. LRRTM4 and GPC4 can induce clustering in each other in a heparan sulfate-dependent manner. PTPσ serves as the presynaptic binding partner with GPC4 and LRRTM4 complex [52]. The complex formed by PTPσ, GPC4, and LRRTM4 functions as trans-synaptic and is essential for excitatory synapse formation and transmission. These findings provide a critical role for GPCs in the structural and functional organization of synapses.

### SPARC (osteonectin)/Hevin (SPARC-like1)

SPARC and Hevin are highly homologous matricellular proteins associated with ECM that regulate ECM synthesis and cell–ECM adhesion [57]. SPARC is a glycoprotein that is highly expressed in the developing CNS. It is expressed by and disappears along with radial glial cells in the early postnatal period. In the adult CNS, it is expressed abundantly in specialized radial glial [58,59]. Hevin is the most strongly expressed ECM component in the CNS [60,61].The expression of Hevin increases in early development after birth, peaks at postnatal day 20, and remains highly expressed in the adult [61]. Hevin is reportedly associated with fibrils containing collagen I [60] and regulates collagen assembly [57]. Hevin is concentrated in the astrocytes around the synaptic cleft in the adult brain [59,62]. SPARC knockout mice revealed more RGC synapse formation in the superior collianlus and enhanced excitatory synaptic function. SPARC also plays an important role in determining the level of AMPA receptors [59,63]. Previous studies have illustrated that it regulates AMPA receptors through β3-integrins and acts as a stabilizer for the GluR2 subunit [64,65]. Consequently, SPARC knockout mice showed an abnormal accumulation of surface AMPA receptors at synapses and impaired synaptic plasticity. Hevin is found to regulate synapse formation by bridging the trans-synaptic neuroligin–neurexin interaction [62]. In Hevin-null mice, the number of synapses in the thalamus cortex is reduced, whereas the expression of SPARC in the hypothalamus and thalamus is selectively increased [62]. Therefore, SPARC and Hevin may play a critical role in regulating the formation of cortical synapses in the thalamus. As SPARC and Hevin show opposite effects on synapse formation, the balance of SPARC and Hevin might be important for normal synaptogenesis and synaptic functioning.

### Neuronal pentraxins

Neuronal activity-regulated pentraxin (NP1/NP2) and neuronal pentraxin receptor (NPR) belong to the long pentraxin superfamily of multifunctional proteins involved in immunological responses [66]. NP1 and NP2 are secreted to the synaptic cleft and present in the ECM as a multimeric extracellular scaffold complex, while NPR is a transmembrane protein [18,26,67,68]. They are both expressed in the cerebral cortex, cerebellum, and hippocampus, especially in CA3 and DG regions. Neuronal pentraxins are enriched in excitatory synapses [69]. Neuronal pentraxins can accumulate AMPA receptors through direct interactions with AMPA receptors [70,71]. The application of exogenous NP2 can induce the accumulation of AMPA receptors in cultured hippocampal neurons [68]. When neural activity occurs, NP2 expression is upregulated, which indicates that it may be involved in activity-dependent synapse formation [68]. These findings indicate that pentraxins participate in postsynaptic specialization by accumulating AMPA receptors during synapse formation.

### C1q-like (C1QL) proteins

C1QL family proteins consist of four members C1QL1–4 that have collagenous triple-helical domains as complement C1q [72,73]. The collagenous triple-helical domains share a feature with collagens [18]. The complement C1q can form a collagen-like structure and regulate ECM remodeling during wound healing [74]. Other proteins containing C1q-like domains, such as CTRPs, are reported to regulate ECM production [75]. Although there is no report that C1QL regulates ECM, C1QL proteins are reported to be located at the synaptic cleft and organize the trans-synaptic complex, which is involved in crucial neuronal processes in various brain regions. Brain-specific angiogenesis inhibitor 3 (BAI3), an adhesion-type G-protein coupled receptor, was found to be the receptor of C1QL1 [76]. C1QL1 and BAI3 are highly expressed in the synaptic cleft between climbing fibers (CF) and Purkinje cells in the cerebellum. The knockout of C1QL1 or BAI3 significantly reduces the synapse number between CF and Purkinje cells and causes impaired motor learning in mice [77,78]. The overexpression of C1QL1 in adult C1QL-null mice can rescue the phenotype, suggesting that C1QL1 functions throughout the lifetime. C1QL2 and C1QL3 are mainly expressed in the synaptic cleft between mossy fibers (MF) and CA3 neurons. Unlike C1QL1, the deletion of C1QL2 or C1QL3 did not influence the synapse number or synaptic structure. However, since C1Ql2 and C1QL3 interact with the GluK2 and GluK4 subunits of postsynaptic kainate receptors (KARs), the recruitment of KARs in the postsynaptic site was abnormal in C1QL2 or C1Ql3 knockout mouse [79], C1Ql2 and C1Ql3 were also reported to bind to neurexin3 at the presynaptic site [79]. Thus, the trans-synaptic complexes composed of neurexin3-C1QL2/3-KARs might be important for the function of the MF–CA3 synapse. These findings suggest that C1QLs serve as the linker of pre- and post-synaptic molecules, which is significant for the recognition between pre- and post-synaptic terminals.

### Precerebellin (Cbln)

Cbln is first identified as a precursor of cerebellin [80].The Cbln family is a member of the C1q family, which has a conserved C1q domain at the C-terminus [81]. The Cbln family has four members: Cbln1–4. Cbln1 is expressed in the cerebellum and secreted by granule cells [82]. The secreted Cblns are located at the synaptic cleft and stabilize trans-synaptic cell adhesion [83]. Cbln1 knockout mice showed a severe decrease in synapse numbers between the parallel fibers and Purkinje cells [84]. When cultured Cbln1-KO Purkinje cells were treated with recombinant Cbln1 in vitro, the synapse number and synaptic transmission were considerably rescued [85]. The in vivo injection of recombinant Cbln1 into Cbln1 knockout mice rescues these phenotypes [85]. Cbln3 can form a heteromer with Cbln1 and reduce the surface delivery of Cbln1. Accordingly, Cbln3 has been considered a native inhibitor of Cbln1 [86,87]. The function of Cbln1 in synapse formation depends on its trans-synaptic interaction between GluD2 and neurexins [88]. GluD2 is an ionotropic glutamate receptor localized at the postsynaptic site in Purkinje cells that interacts with hexameric Cbln1, which also binds to presynaptic neurexins. Cbln1 can bridge the GluD2/neurexin trans-synaptic complex in the synapse organization of Purkinje cells [89,90]. Cbln1 can also stabilize GluD2 at postsynaptic sites and induce presynaptic differentiation by binding with neurexins at the presynaptic site. These findings suggest that Cblns serve as the linker of pre- and post-synaptic molecules to drive synapse formation.

### Lgi1

Lgi1 is one of the secretory proteins containing the leucine-rich repeat (LRR) domain [91]. The secreted Lgi1 serves as a scaffold protein at the synaptic cleft [18]. Lgi1 can form a dimer though its LRR domain and interact with ADAM22 proteins at both pre- and post-synaptic sites [91,92]. The Lgi-ADAM22 complex was reported to interact with MAGUKs at the pre- or post-synaptic site and Kv1 (Kcna) channels at the presynaptic site [93]. The pre-and post-synaptic MAGUKs further recruit voltage-dependent Ca^2+^ channels, AMPA and NMDA receptors, and cell adhesion molecules to form trans-synaptic nanocolumns [94]. The depletion of Lgi1 or ADAM22 disrupts the nanocolumns and epileptic phenotypes in mice [95]. Recently, the mutation of Lgi1 and the auto-antibody of Lgi1 have been reported to lead to neuronal diseases, such as seizure, epilepsy, and cognitive amnesia [96,97]. Accordingly, it is worth examining Lgi1 as a therapeutic target in future work.

### Agrins

Agrin, a kind of HSPG, was identified as a synaptic ECM protein and was found to play a critical role in postsynaptic differentiation at neuromuscular junctions (NMJ) [26,98,99]. During NMJ formation, the nerve terminal releases agrin, which is stabilized in the basement membrane. Agrin can activate MuSK at a postsynaptic site and cluster acetylcholine receptors (AChRs) at a postsynaptic site [100]. Several studies have reported that agrin is also expressed in the CNS and regulates synapse formation and synaptic function. Unlike serving as an organizer in NMJ, agrin mainly serves as a signaling transduction molecule in the CNS. Agrin can induce specific responses in hippocampal neurons and cause the phosphorylation of transcription factor CREB to induce the transcription of its downstream genes. The application of agrin in cultured cortical neurons induces the expression of immediate-early gene c-fos expression [101,102]. The specific receptor of agrin in the CNS was found to be alpha3NKA, a member of the Na+/K+-ATPase (NKA) family. Agrin binds to alpha3NKA and inhibits its pump activity, modulating the membrane and action potential of neurons. Depleting agrin with antisense oligonucleotides or specific antibodies impairs dendrite development and the loss of synapse numbers. These findings suggest the critical role of agrin in synaptogenesis and synapse formation in the CNS besides NMJ.

### Laminins

Laminins are key components of the ECM in the NMJ. Laminins form large, multi-armed glycoproteins with α, β, and γ chains [103]. Thus far, five kinds of α, three kinds of β, and three kinds of γ have been identified [104]. Lamin α, β, and γ form the heterotrimer extracellular matrix and play a critical role in synaptogenesis by guiding cell differentiation, cell migration, and cell adhesion [105]. Different combinations of laminin chains are at various regions of NMJ: The α2β2γ1 laminin is mainly located at the basal lamina in the extra-synaptic site of NMJ, while α2β2γ1, α4β2γ1, and α5β2γ1 are located at the synaptic cleft of the NMJ [106]. Laminins are essential for NMJ development and maintenance and presynaptic organization. The knockout of γ1 is lethal due to the impairment of endoderm differentiation [107]. In addition, the knockout of β2 was lethal to mice 15–30 days after birth [108]. The NMJ in β2 knockout mice reveals abnormal pre- and postsynaptic terminals and junction folds, as well as synaptic adhesion, which leads to reduced neurotransmitter release and decreased synaptic transmission [108–110]. The deletion of α4 in mice does not influence the number of presynaptic active zones and junction folds, but impairs the trans-synaptic alignment of the NMJ structure [111]. The deletion of α5 arrests postsynaptic maturation [112]. Moreover the ablation of α2 causes abnormalities in the muscle endplates but not the active zone [113,114]. Laminins have also been reported to regulate synapse formation in the CNS [115]. The knockout of Laminin α5 causes the loss of spine numbers and abnormal spine morphology [116]. These findings suggest a critical role for laminins in both NMJ and CNS synapse organization and formation.

### Collagens

Collagens are important components of the basement membrane, a specialized ECM in many tissues [117]. Moreover, 28 types of collagens are found (I–XXVIII) [117]. In the NMJ, the collagens are critical components of the basal lamina, which are involved in normal NMJ development, differentiation, and stabilization [118]. The deletion of collagen IV causes axon defects and abnormal presynaptic specialization in the NMJ [119]. The ablation of collagen VI induces defects in the endplate in NMJ [120]. In addition, in the NMJ, some collagens are reportedly located at the CNS and regulate synaptogenesis. Detected in the cerebellum and expressed by Purkinje cells, collagen XVIII is critical for synapse formation between climbing fiber axons and Purkinje cell dendrites [121]. The ablation of collagen XVIII significantly reduces synapse numbers. Collagen XIX has also been reported to be a synaptic organizer in the hippocampus [122]. It is expressed by Gad67-positive interneurons and is essential for the inhibitory synapse formation of synaptotagmin-2-containing nerve terminals in the subiculum.

### Nidogens

Nidogens are one of the major components of basement membranes. Reported to interconnect collagens and laminins, nidogens maintain the integrity of basement membranes in NMJ [118]. Nidogen-1 is located at the basal lamina, while nidogen-2 is located at the synaptic site. The knockout of nidogen-2 in mice shows a normal NMJ structure at birth, but abnormal postsynaptic architecture with immature and fragmented AChRs in adults [123]. Nidogen-1 has been found to play a role in the hippocampal synaptic function [122], and the ablation of nidogen-1 causes increased excitability and the loss of perforant-path long-term synaptic potentiation in the CA1 and dentate gyrus of the hippocampus while revealing no morphological changes. These works suggest that although the synapse in the CNS lacks a basement membrane, the components of the basement membrane can regulate synapse organization and specialization.

### Pikachurin

Pikachurin, another kind of HSPG, was identified as an extracellular matrix-like protein in the synaptic cleft of photoreceptor ribbon synapses [124]. Pikachurin is released by photoreceptors and coordinates with the presynaptic dystroglycan glycoprotein complex. The pikachurin–dystroglycan complex recruits downstream ON-bipolar neurons by interacting with GPR179 at a postsynaptic site [124,125]. This trans-synaptic assembly plays a notable role in the synaptic transmission of photoreceptor signals. The ablation of pikachurin significantly affects the synapse formation and synaptic transmission of photoreceptor ribbon synapses, as well as visual functions in mice. The finding of pikachurin’s regulation on the formation of photoreceptor ribbon synapses suggests the specific ECM organizer of specific synapses.

## ECM components participate in synapse development and maturation

### Tenascin (Tn)

Tn family members mainly include four types: Tn-C, Tn-X, Tn-R, and Tn-W. Tn-C is assembled as a hexamer through a disulfide link [126,127]. In the developing CNS, Tn-C is downregulated with adulthood, suggesting that Tn-C-related proteins may play a role in early development [128]. Previous studies have demonstrated that Tn-C can promote axon outgrowth and affect cell migration, synapse formation, and synaptic plasticity in adult organisms. The hippocampus is a characteristic area of Tn-C expression in the brain. In the Tn-C-null mouse model, the number of somatostain-positive interneurons in the hippocampus was reduced, and short-term plasticity was changed. In electrophysiological recordings of the Tn-C-null mouse cerebellar slice [129], short-term plasticity is also changed. These changes may be mediated by the regulation of Tn-C on L-type voltage-dependent Ca^2+^ channels [130]. Tn-R is synthesized by oligodendrocytes during the formation of myelin sheaths and is abundant in the neural network surrounding inhibitory interneurons [131]. In Tn-R-deficient mice, TBS-induced LTP decreases, and the basic excitatory synaptic transmission in the hippocampal CA1 area is enhanced [132]. This phenotype is due to the loss of Tn-R, which causes the deinhibition of the hippocampal CA1 area and the change of threshold in LTP induction [133,134]. These findings suggest the extensive role of tenascin in synapse development and function.

### Hyaluronan (HA)

HA is a glycosaminoglycan chain containing repeated disaccharides, and it is gathered on the cellular membrane by transmembrane HA synthases [135]. HA can bind to the lectican family, such as aggrecan, neurocan, versican, and brevican [136], and regulate the synaptic structure through these interactions. HA is reported as a component of perineuronal nets (PNN), that is, extracellular matrix structures that surround neurons and regulate neuroplasticity and memory [137]. Link proteins can stabilize the binding of hyaluronan to aggrecan and are involved in PNN formation [138]. HA plays different roles in different stages of neural development and can regulate neuron migration, neural precursor cell proliferation, and neuron differentiation and maturation [139]. Recent work has found that HA can affect synapse formation. The HA level is high around the nascent excitatory synapse, and high levels of HA suppress the formation of synapses, while the removal of HA increases the density of excitatory synapses [140]. HA-based ECM has also been reported to regulate the surface distribution of glutamate receptors. The removal of HA with hyaluronidase increases the lateral diffusion of AMPA receptors and modulates the short-term plasticity of neurons [141]. The removal of HA can also induce the GluN2 subunit composition of NMDA receptors to switch from GluN2A to GluN2B [142]. These findings provide a critical role for HA in synapse formation and synaptic transmission.

### Reelin

Reelin is secreted by GABAergic interneurons [143]. It is a large ECM glycoprotein containing an N-terminal domain that is similar to F-spondin, eight reelin repeats that contain an EGF-like repeat, and a C-terminus that contains a highly basic domain [144]. Reelin was found to play critical roles in mediating neuronal circuit formation via promoting synapse maturation, shaping, and stabilizing dendrites and spines [145–147]. The overexpression of reelin in the postnatal mouse forebrain increases spine head size and promotes synapse formation [148]. During hippocampal maturation, reelin helps control the subunit composition of NMDA receptors. Very low-density lipoprotein receptors (VLDLR) and ApoE receptors have been found to receive reelins [149]. VLDLR and ApoE2 can interact with NMDA receptors through PSD95. The chronic suppression of reelin’s function could abolish the decrease in NR2B-dependent NMDAR-mediated currents, which is a typical characteristic of mature synapses [150]. As reelin is highly associated with NMDA receptors, reelin signaling has also been reported to regulate synaptic plasticity [150]. These findings suggest that reelin regulates postsynaptic specialization via the regulation of NMDAR functions.

### Chondroitin sulfate proteoglycans (CSPGs)

CSPGs are ECM components present in both the developing and adult CNS [151]. They include leucine-rich CSPGs, phosphocan, and four members of the lectican family (i.e., aggrecan, neurocan, versican, and brevican) [152]. In the adult nervous system, CSPGs are highly expressed in the perineuronal nets (PNN) and play a role in stabilizing synapses [153]. Using chondroitinase ABC (ChABC) to digest CSPGs in hippocampal slices increases the mobility of dendritic spines, thus causing abnormal spine head protrusions [154,155]. The change of dendritic spines is associated with integrin β1 receptors’ activation and focal adhesion kinase at synaptic sites [156]. CSPGs also affect synaptic plasticity, and mice lacking CSPGs depicted impaired LTP. The removal of CSPGs has been reported to cause the degradation of PNN, heightening the excitability of perisomatic interneurons and suppressing LTP induction [157]. Such findings indicate that CSPGs are involved in activity-dependent synapse formation.

### Matrix metalloproteinases

Matrix metalloproteinases (MMP) are defined by their function in cleaving and remodeling the ECM [158]. Currently, 24 MMPs have been identified. Several MMPs are reportedly expressed in the brain, and they play a role in synaptogenesis in physiological and pathological conditions. The expression of MMP3 and MMP9 is increased with kainate (KA)-induced excitotoxicity in the granular neurons of the dentate gyrus, which causes the reactive synaptogenesis and development of LTP after injury [159–162]. Additionally, the knockout of MMP9 disrupts the LTP induction, learning, and memory of mice [160]. The expression of MMP3 and MMP9 was transiently increased during water maze experiments [160]. MMP7 has been reported to impair dendritic spines in the hippocampus. It induces mature mushroom spines to transform into thin filopodia spines [163]. MT5-MMP was found to be enriched in the synaptosome of hippocampal neurons and to remodel synapses’ structure by cleaving cell adhesion molecule N-cadherin [164].

## ECM in neurological diseases

### ECM and Alzheimer’s disease

Changes in various ECM components are associated with Alzheimer’s disease (AD) through different mechanisms. The various components of the ECM can limit the process of certain diseases and promote the development of certain diseases. In AD, the expression profiles of ECM components changes. For instance, HA, CSPG, and tenascin are up-regulated, and reelin is down-regulated in AD [165]. The abnormal expression of HA might cause the demyelination of neurons, which leads to impaired nerve signaling transmission [166,167]. Another study reported that heightened HA is associated with vascular injury in AD, which leads to a lack of oxygen and glucose in the brain and further causes neuronal damage and synapse loss [168]. Aβ plaques are the typical marker of AD in the brain [169]. The up-regulation of CSPGs and tenascin-R, the main components of PNN, causes high stability in PNN [170,171]. Stable PNN was found to surround Aβ plaques and protect them from degradation [172]. In addition, overstabilized PNN inhibits the growth of neurites and restricts the signaling transmission between neurons and synaptic plasticity [173,174]. Other work has reported the remodeling of PNN glycan structures in AD [175]. The chondroitin-sulfate glycosaminoglycan (CS-GAG), which is attached to the CSPG proteins in PNN, illustrates sulfation modifications in AD and results in the remodeling of PNN [175]. The treatment of AD model mice with ChABC reduces Aβ plaques' deposition, restores synaptic density, and alleviates AD symptoms, such as LTP impairment and hippocampus-dependent memory loss [176]. The upregulation of tenascin-C has been reported to be associated with inflammatory activation in AD [177]. Moreover, tenascin-C can cause chronic inflammation via pro-inflammatory cytokines, so reducing tenascin-C significantly decreased the activity of secretase in the hippocampus and cortex of AD mice, therefore reducing synapse damage in AD [178]. Unlike the up-regulation of most components of the ECM, reelin was found to be depleted in the progress of AD [179], even before the onset of Aβ plaque deposition. It has been proven that reelin can inhibit the production of Aβ and promote the clearance of Aβ [180]. The phosphorylation of Tau protein is another pathological marker of AD. Reelin also has been reported to prevent the phosphorylation of Tau by inhibiting the expression of GSK-3β, the most effective Tau protein kinase [181]. These findings suggest that reelin has a protective effect against AD.

### ECM and schizophrenia

Schizophrenia (Sz) is a severe brain disorder with clinical symptoms, including hallucinations, delusions, affective flattening, and cognitive deficits [182]. It is believed that neural circuit development and maturation impairment might contribute to Sz [182]. Several studies have reported that the expression patterns of ECM components are associated with Sz [183–185]. For instance, CSPG-labeled PNNs decreased in the amygdala, entorhinal cortex, layers 3 and 5 of the prefrontal cortex, and olfactory receptor neurons (ORNs) of subjects with Sz [183–185]. Decreased PNN might cause unstable synapses, leading to impaired synaptic pruning and prolonged neural plasticity [182]. Another work reported that lessened PNN might lead to the dysfunction of GABAergic interneurons [184]. The expression of reelin was also reduced in the temporal cortex, prefrontal cortex, caudate nucleus, and hippocampus in patients with SZ [186]. Reduced reelin in SZ was reported to cause abnormal GABA-mediated synaptic transmission by reducing the GABA-synthesizing enzyme GAD67 [182]. As reelin is an important regulator of the subunit composition of NMDA receptors, decreased reelin also affects excitatory synaptic transmissions [187]. These effects might lead to an imbalance between excitatory and inhibitory synaptic transmisson, which causes dysfunction in patients with Sz.

## Abbreviations

AD: Alzheimer’s disease
AMPA: α-amino-3-hydroxy-5-methyl-4-isoxazole propionic acid
CAM: cell adhesion molecule
ChABC: chondroitinase ABC
CNS: central nervous system
CS-GAG: chondroitin-sulfate glycosaminoglycan
ECM: extracellular matrix
GPI: glycosyl phosphoinositide
HA: hyaluronan
HSPG: heparan sulfate proteoglycan
LRR: leucine-rich repeat
NPR: neuronal pentraxin receptor
PNN: perineuronal net
VLDLR: very low-density lipoprotein receptors
